# Generational Influence on Diet Quality: An Analysis Based on Vigitel 2023

**DOI:** 10.1111/jhn.70320

**Published:** 2026-07-29

**Authors:** João Paulo Lima de Oliveira, Laudicéia Ferreira Fróis, Bianca Aparecida de Sousa, Hemily Lopes Menezes Silvério, Giovana Oliveira Mendonça, Leticia Ohara de Paiva, Nathalia Luiza Ferreira, Lílian Gonçalves Teixeira

**Affiliations:** ^1^ Department of Nutrition, Faculty of Health Sciences Federal University of Lavras Lavras Minas Gerais Brazil; ^2^ School of Nutrition Federal University of Ouro Preto Ouro Preto Minas Gerais Brazil; ^3^ Department of Medicine, School of Health Sciences Federal University of Lavras Lavras Minas Gerais Brazil

**Keywords:** age, dietary surveys, nutrition policy, population surveillance, ultra‐processed foods

## Abstract

**Background:**

Food consumption is influenced by sociodemographic factors throughout life, including generational affiliation. Different cohorts, such as Traditionalists, Baby Boomers, Generation X, Generation Y and Generation Z, are considered. In this context, the study aimed to examine the association between generational groups and diet quality, specifically the consumption of unprocessed/minimally processed foods and ultra‐processed foods (UPF), using data from the 2023 Vigitel survey in Brazil.

**Methods:**

This is a population‐based cross‐sectional study conducted using data from VIGITEL 2023, including adults (≥ 18 years) residing in Brazilian state capitals. Dietary intake, the outcome variable, was assessed using questions based on the VIGITEL questionnaire, followed by the construction of a score considering consumption frequency and the level of food processing. Generational status was defined based on the difference between participants' reported age and the year of data collection, with individuals classified into the following cohorts. Traditionalists, Baby Boomers, Generation X, Generation Y and Generation Z. Univariate and multivariable logistic regression models were used to assess associations, adjusting for educational level, marital status, and geographic region, with statistical significance set at *p* < 0.05.

**Results:**

The final sample comprised 21,690 Brazilian adults. Individuals from Generation X (OR: 1.78; 95% CI: 1.33–2.38), Generation Y (OR: 3.24; 95% CI: 2.42–4.34), and Generation Z (OR: 3.45; 95% CI: 2.46–4.83) exhibited significantly higher odds of consuming a lower‐quality diet compared to Traditionalists.

**Conclusion:**

Younger generations demonstrated a greater propensity toward diets high in ultra‐processed foods, characterised by elevated energy density, added sugars, and saturated fats, underscoring the need for targeted public health strategies.

## Summary

- The prevalence of a low‐quality diet was higher among men.
- Generation Z is more likely to consume a lower‐quality diet.
- Younger generations are more likely to have a lower‐quality diet than the Traditionalist generation.
- Generations X, Y and Z are more susceptible to consuming ultra‐processed foods.

## 1 Introduction

In recent decades, transformations in food systems have substantially reshaped population eating behaviours, with direct implications for diet quality and health outcomes. In this context, a progressive shift has been observed from the consumption of fresh and minimally processed foods toward industrialised products with a higher degree of processing, particularly ultra‐processed foods (UPF) [1, 2]. In Brazil, this process is reflected in the increasing contribution of UPF to population food intake, accompanied by a reduction in the consumption of fresh and minimally processed foods [1, 2]. This transition represents a structural reconfiguration of food consumption, characterised by higher energy density and lower fibre content [3, 4, 5], and is associated with an increased burden of non‐communicable diseases (NCDs). This phenomenon is not evenly distributed, showing variation across generations, which highlights the influence of historical and social contexts in shaping food consumption over time [1, 2, 6]. From a health perspective, dietary quality is a key determinant of population health outcomes. Higher consumption of fruits, vegetables, legumes, and whole grains has been associated with reduced risk of obesity, improved metabolic health, and increased longevity [7]. In parallel, highe UPF has been associated with adverse health outcomes, including a dose–response relationship with obesity [8, 9, 10], and increased risk of cardiovascular disease, type 2 diabetes, and certain cancers, independent of adiposity [11, 12, 13, 14]. The concept of generation refers to groups of individuals who share similar historical and social experiences that shape values, practices, and lifestyles [15, 16]. These shared experiences contribute to differences in food consumption across cohorts, as changes in the food environment, food availability, and everyday life organisation directly influence dietary choices [17]. Evidence suggests that younger individuals tend to show higher consumption of ready‐to‐eat foods, whereas older individuals maintain a greater frequency of home‐cooked meals [18, 19, 20], reflecting both transformations in the food system and contemporary social dynamics. This pattern has also been observed in international contexts. In China, lower consumption of staple foods and vegetables has been identified among younger generations, alongside higher intake of animal‐source foods and fats [21]. In Canada, ultra‐processed food intake is widely prevalent, with differences according to sociodemographic characteristics, being higher among younger individuals, men, and those with lower educational attainment [22]. These findings reinforce that changes in food consumption are embedded in broader processes of food system transformation, driven by globalisation, industrialisation of food production, and the accelerating pace of contemporary life [23, 24]. These changes in food consumption tend to be established early in life and persist across the life course [25, 26, 27]. Notably, higher consumption of fresh and minimally processed foods is observed among older adults, particularly from the age of 50 onward [28, 29]. This pattern also varies according to sociodemographic characteristics, being more common among women and individuals who perceive their income as sufficient to cover household expenses [30]. In Brazil, the Surveillance System for Risk and Protective Factors for Chronic Diseases by Telephone Survey (Vigitel) is one of the main sources for monitoring population food intake and has been widely used in public health research [6, 31, 32]. Despite advances in the literature on food consumption in the country, studies examining generational differences in population‐based samples remain scarce. Given this gap, analysing variations in food consumption across generations may help elucidate how social and historical transformations are reflected in the foods consumed by different age groups. In this sense, the present study aimed to analyse the association between the consumption of fresh and minimally processed foods and UPF across different generations in Brazil, using data from Vigitel 2023.

## 2 Methods

The data analysed in this study were obtained from the 2023 edition of the Vigitel, a cross‐sectional survey designed to monitor the frequency and distribution of key determinants of NCDs in Brazil.

Vigitel employs a two‐stage cluster sampling design. In the first stage, residential landlines and mobile phone numbers are randomly selected. In the second stage, one adult (≥ 18 years old) residing in the selected household is randomly chosen to complete the standardised questionnaire. The sample size is adjusted for design effect and variability to ensure accurate representation of the adult population living in the 26 Brazilian state capitals and the Federal District [6].

Dietary intake was assessed using self‐reported dichotomous (yes/no) information on food consumption on the day prior to the interview. No information on portion sizes or consumption frequency was available, which precluded the use of more detailed quantitative dietary assessment methods. Therefore, an exploratory unweighted binary dietary score was constructed to summarise dietary patterns at the individual level. Each food item was assigned equal weight as a simplifying assumption, given the absence of information on portion sizes or consumption frequency and the objective of ranking individuals according to their relative dietary patterns rather than estimating the nutritional contribution of individual foods. Accordingly, the score should be interpreted as an exploratory, non‐validated indicator of dietary patterns rather than as a validated measure of overall diet quality or nutrient intake. A total of 24 food items were evaluated and classified into two groups: 12 unprocessed or minimally processed foods and 12 ultra‐processed foods (UPFs). Food classification followed the Dietary Guidelines for the Brazilian Population and the NOVA food classification system, which categorises foods according to the extent and purpose of industrial processing [33].

The group of unprocessed/minimally processed foods included: leafy greens (lettuce, kale, broccoli, watercress, or spinach); root and tuber vegetables (pumpkin, carrot, sweet potato, okra or caruru); fresh fruits (papaya, mango, yellow melon, or pequi); vegetables (tomato, cucumber, zucchini, eggplant, chayote or beet); citrus and other fruits (orange, banana, apple or pineapple); grains and cereals (rice, pasta, cornmeal, couscous or green corn); legumes (beans, peas, lentils or chickpeas); starchy roots (white potato, cassava, yam or taro); fresh meats (beef, pork, chicken or fish); eggs (fried, boiled or scrambled); milk; and nuts (peanuts, cashew nuts or Brazil nuts). The group of UPFs included: soda; boxed/canned fruit juice; powdered drink mixes; chocolate‐flavoured milk beverages; flavoured yogurt; packaged salty snacks or crackers; sweet cookies, filled biscuits, or packaged cakes; chocolate, ice cream, gelatin, pudding or other processed desserts; processed meats (sausage, bologna, mortadella or ham); industrial bread (sandwich bread, hot dog buns or hamburger buns); margarine; and instant noodles, powdered soups, frozen lasagna or other ready‐to‐eat frozen meals [6].

The dietary intake score was calculated by assigning a value of 0 or 1 to each food item. Foods classified as unprocessed or minimally processed received a score of 1 when they were not consumed on the previous day and 0 when they were consumed. Conversely, UPFs received a score of 1 when consumed and 0 when not consumed. Therefore, higher scores represented dietary patterns characterised by lower consumption of unprocessed or minimally processed foods and greater consumption of UPFs, whereas lower scores indicated greater consumption of unprocessed or minimally processed foods and lower consumption of UPFs [34, 35, 36].

For analytical purposes, the overall dietary intake score was categorised according to its distribution in the study sample. Because no established cutoff is available for this exploratory score, the 75th percentile was used to identify participants with less favourable dietary patterns. Participants with scores below the 75th percentile were classified as having a higher‐quality diet, whereas those with scores equal to or above the 75th percentile were classified as having a lower‐quality diet [34, 35, 36].

Participants' generational status was classified based on age, derived from the self‐reported date of birth. Age was calculated as the difference between the reported date of birth and the study year (2023). Participants were then categorised into the following generational groups: Traditionalists (T) (born between 1934 and 1945), Baby Boomers (BB) (1946–1964), Generation X (1965–1980), Generation Y (1981–1998) and Generation Z (1999–2019) [16]. Individuals born before 1934 were excluded, as the sampling design was restricted to participants aged up to 89 years (*n* = 102) in 2023.

Sociodemographic variables such as sex (male, female), race/skin colour (White, Black/Brown, Yellow/Indigenous), educational attainment (illiterate, ≤ 9 years, > 9 years of schooling), marital status (with partner, without partner), and region of residence (North, Northeast, Central‐West, Southeast, South) were obtained through standardised Vigitel 2023 questionnaires [6].

Descriptive analyses were performed using relative frequencies and 95% confidence intervals (95% CI). Associations between categorical variables were tested using Pearson's chi‐square test. Associations between variables were examined using univariate and multivariable logistic regression models, with adjustment for education level, marital status, and region of residence. All analyses were conducted using Stata® software, version 13.0 (College Station, Texas, United States), incorporating the svy command to account for the complex survey design. Statistical significance was set at 5%.

The Vigitel study was approved by the National Research Ethics Committee (CONEP) of the Brazilian Ministry of Health (CAAE: 65610017.1.0000.0008).

## 3 Results

The sample comprised 21,690 adults. Male participants were more likely to have lower dietary scores (55.4%). In contrast, higher scores were observed among Generation Y individuals (aged 25–42 years) (45.9%), those who self‐identified as Black or Brown (61.0%), individuals without a partner (58.8%), and residents of the Southeast region (47.9%) (Table 1).

**Table 1:** Sociodemographic characteristics according to food consumption score classification (Vigitel, 2023).

Variables		Food consumption score		
	Total % (95% CI)	< p75* % (95% CI)	≥ p75* % (95% CI)	*p* value
Sex				
Male	54.0 (52.4–55.5)	55.4 (53.4–57.2)	51.8 (49.3–54.4)	**0.029**
Female	46.0 (44.5–47.6)	44.6 (42.8–46.5)	48.2 (45.6–50.7)	
Age (Generational status)				
Generation Z (≤ 24 years)	12.9 (11.8–14.1)	10.5 (9.2–12.1)	16.5 (14.8–18.4)	**< 0.001**
Generation Y (25–42 years)	39.4 (37.9–40.9)	35.1 (33.2–36.9)	45.9 (43.3–48.5)	
Generation X (43–58 years)	27.5 (26.2–28.8)	30.3 (28.7–32.0)	23.3 (21.3–25.4)	
Baby Boomers (59–77 years)	17.1 (16.1–18.0)	20.2 (19.0–21.5)	12.3 (10.9–13.9)	
Traditionalists (78–89 years)	3.1 (2.8–3.5)	3.9 (3.5–4.4)	2.0 (1.6–2.5)	
Race/Skin colour				
White	39.8 (38.0–41.3)	41.7 (39.9–43.6)	36.9 (34.4–39.4)	**0.006**
Black/Brown	58.0 (56.5–59.5)	56.1 (54.2–57.9)	61.0 (58.5–63.5)	
Asian/Indigenous	2.2 (1.8–2.6)	2.2 (1.7–2.9)	2.1 (1.6–2.9)	
Marital status#				
With partner	46.5 (45.0–48.0)	49.5 (47.7–51.4)	42.0 (39.5–44.5)	**< 0.001**
Without partner	53.5 (52.0–55.0)	50.5 (48.7–52.3)	58.0 (55.5–60.5)	
Education level				
Illiterate	1.0 (0.8–1.4)	1.1 (0.7–1.5)	1.0 (0.7–1.5)	0.227
≤ 9 years of schooling	24.8 (23.4–26.1)	23.8 (22.3–25.4)	26.1 (23.7–28.6)	
> 9 years of schooling	74.2 (72.8–75.6)	75.1 (73.5–76.7)	72.9 (70.3–75.3)	
Geographic region				
North	10.7 (10.1–11.2)	10.6 (9.9–11.3)	10.8 (9.8–11.8)	**< 0.001**
Northeast	25.3 (24.3–26.3)	26.9 (25.6–28.2)	22.9 (21.3–24.7)	
Southeast	44.1 (42.5–45.7)	41.6 (39.6–43.6)	47.9 (45.3–50.5)	
South	8.0 (7.5–8.5)	8.0 (7.4–8.7)	8.0 (7.1–8.9)	
Midwest	11.9 (11.3–12.6)	12.9 (12.0–13.9)	10.4 (9.4–11.5)	

Abbreviation: CI, confidence interval.

* < p75: higher‐quality diet; ≥ p75: lower‐quality diet.

^#^ With partner: married or in a stable union for more than six months; without partner: widowed, divorced, or single. Bold values indicate statistical significance (*p*‐value < 0.05). Pearson's chi‐square test.

**Figure 1:**
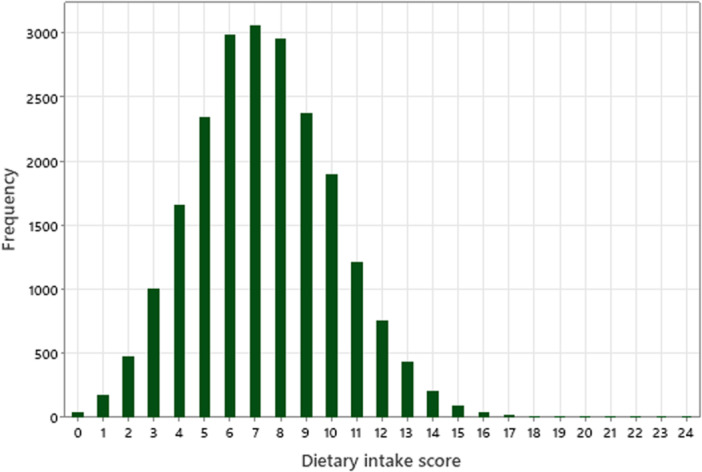
Frequency distribution of the dietary intake score (Vigitel, 2023).

The distribution of the food consumption score showed a median of 7 points, ranging from 0 to 19 points, as illustrated in Figure 1.

In the association analysis, younger generations were found to have significantly higher odds of presenting poorer dietary quality compared to the Traditionalist generation. The odds of a lower‐quality diet were 1.78 times higher among Generation X (OR: 1.78; 95% CI: 1.33–2.38), 3.24 times higher among Generation Y (OR: 3.24; 95% CI: 2.42–4.34), and 3.45 times higher among Generation Z (OR: 3.45; 95% CI: 2.46–4.83), as shown in Table 2.

**Table 2:** Association between food consumption score classification and generational status (Vigitel, 2023).

Food consumption score						
	Unadjusted analysis			Adjusted analysis*		
Variables	OR	95% CI	*p* value	OR	95% CI	*p* value
Gerational status						
Traditionalist	1.00	—	—	1.00	—	—
Baby boomer	1.18	0.90–1.56	0.228	1.31	0.98–1.74	0.068
Generation X	1.49	1.15–1.94	**0.003**	1.78	1.33–2.38	**< 0.001**
Generation Y	2.54	1.96–3.29	**< 0.001**	3.24	2.42–4.34	**< 0.001**
Generation Z	3.03	2.24–4.11	**< 0.001**	3.45	2.46–4.83	**< 0.001**

Abbreviations: CI, confidence interval; OR, odds ratio.

* Adjusted for age, education level, marital status, and macro‐region of residence; Bold values indicate statistical significance (*p*‐value < 0.05).

## 4 Discussion

The present study stands out for examining generational differences in food consumption using a large population‐based survey in Brazil, showing a higher likelihood of lower‐quality diet among Generation X, Y and Z compared with the Traditionalist generation. Similar age‐ and generation‐related gradients, characterised by poorer dietary profiles among younger adults, have also been reported in national surveys and population‐based studies [15, 16, 37, 38]. The generational differences observed in this study suggest differences in food consumption across age groups. The higher contribution of UPFs among younger individuals and the greater consumption of fresh or minimally processed foods among older generations [15] indicate a heterogeneous distribution of diet quality across populations. In addition, variation across sociodemographic markers reinforces that these differences are associated with social conditions that structure access to and organisation of dietary intake [39]. In contrast, older individuals tend to report greater health concerns, often associated with the presence of NCDs, which may be related to more favourable food choices in this group [29, 40, 41]. In this context, the life course perspective provides an interpretative framework for understanding these patterns by considering dietary intake as a behaviour shaped by different experiences and social contexts over time. This framework highlights the relevance of factors such as childhood eating practices, changes in the family environment, socioeconomic conditions, and food availability, which help interpret the differences observed between generational groups [42, 43]. In addition to structural factors, differences in food literacy and culinary skills also contribute to understanding these findings. Lower levels of these competencies may favour convenience‐based choices, increasing reliance on UPFs [44]. Although these variables were not directly assessed in the present study, the lower‐quality diet profile observed among younger generations may reflect broader contemporary food environment influences and lifestyle characteristics. Supporting this perspective, evidence among adolescents and young adults has shown that lower cooking skills are associated with higher UPF consumption and lower overall diet quality [45]. Additionally, greater time constraints among younger generations, who are more likely to combine work, study, and family responsibilities, may contribute to increased reliance on convenience foods and higher UPF consumption. Although not directly assessed in the present study, this represents a plausible contextual factor influencing dietary patterns across generations. In this context, understanding dietary behaviours across generations is relevant for public health. Addressing these determinants is essential for developing strategies that promote healthier and more sustainable dietary patterns, particularly in light of concerns regarding environmental sustainability, population health, and food security. Interventions targeting younger generations, especially Generation Z, should emphasise awareness of the impacts of food choices and encourage healthier and more accessible eating practices [46]. This study is among the first to examine generational food consumption using Brazil's largest population‐based survey. In this sense, the findings provide relevant evidence for public health interventions. From a life course perspective, strategies should prioritise early stages of life, considering that dietary behaviours tend to persist over time, as described in the literature [25, 26, 27]. In addition, improving food literacy and strengthening culinary skills may help reduce reliance on UPFs, particularly among younger individuals [47, 48]. Beyond individual‐level interventions, structural actions are also needed, such as policies aimed at reducing the availability and marketing of UPFs while improving access to fresh or minimally processed foods. Together, these strategies may help reduce observed generational disparities and their potential health consequences. Finally, some limitations should be considered. Dietary intake was assessed using a single self‐reported measure referring to the previous day, which does not allow the estimation of habitual intake and is subject to recall bias. In addition, the dietary information was limited to dichotomous (yes/no) responses and did not include consumption frequency or portion sizes, precluding the use of more detailed dietary assessment methods and validated diet quality indices. Consequently, the exploratory unweighted binary dietary score used in this study should be interpreted as a relative indicator of dietary patterns rather than a validated measure of overall diet quality, as it does not account for the quantity consumed or the differential contribution of specific foods. Finally, the cross‐sectional design precludes causal inferences, allowing only the identification of associations. However, as the study was conducted via telephone interview, participation was contingent upon having an active telephone line, which may have limited the representativeness of individuals lacking access to this resource.

## 5 Conclusion

The present study identified generational differences in food consumption, with lower‐quality diet profiles among Generation X, Y and Z compared with the Traditionalist generation. These findings indicate distinct dietary patterns across generational groups within a context of broader social, economic, and food system changes. The higher contribution of energy‐dense foods among younger generations suggests consistent differences in dietary profiles across age groups, reinforcing the importance of strategies aimed at promoting healthier food environments and strengthening food literacy at earlier stages of life. Future studies using longitudinal designs are recommended to better characterise changes in dietary intake over time and to further explore the social and environmental determinants underlying the observed differences across generations.

## Author Contributions

**João Paulo Lima de Oliveira:** conceptualisation, investigation, methodology, data curation, formal analysis, writing – original draft, writing – review and editing. **Laudicéia Ferreira Fróis:** conceptualisation, investigation, methodology, formal analysis, writing – original draft. **Bianca Aparecida de Sousa:** conceptualisation, methodology, writing – original draft. **Hemily Lopes Menezes Silvério:** investigation, data curation. **Giovana Oliveira Mendonça:** investigation, writing – original draft. **Leticia Ohara de Paiva:** investigation; writing – original draft. **Nathalia Luiza Ferreira:** methodology, supervision, writing – review and editing. **Lílian Gonçalves Teixeira:** conceptualisation, supervision, writing – original draft, writing – review and editing.

## Funding

The authors have nothing to report.

## Conflicts of Interest

The authors declare no conflicts of interest.

## 1 Peer Review

For transparency, the peer review documents associated with this article are available at http://doi.org/10.1111/jhn.70320.

## Data Availability

The data that support the findings of this study are available from the corresponding author upon reasonable request.
